# Global Protection and the Health Impact of Migration Interception

**DOI:** 10.1371/journal.pmed.1001038

**Published:** 2011-06-14

**Authors:** Zachary Steel, Belinda J. Liddell, Catherine R. Bateman-Steel, Anthony B. Zwi

**Affiliations:** 1 Centre for Population Mental Health Research, South West Sydney Area Health, and Psychiatry Research and Teaching Unit, School of Psychiatry, University of New South Wales, Sydney, Australia; 2 GlobalHealth@UNSW, School of Public Health and Community Medicine, The University of New South Wales, Sydney, Australia

## Abstract

In the fourth article in a six-part *PLoS Medicine* series on Migration & Health, Zachary Steel and colleagues discuss the interception phase of migration and the specific health risks and policy needs associated with this phase.

**Summary Points**

- The volume of international travel and irregular migration places pressure on states to maintain orderly migration programs. Interception strategies are increasingly used by states to halt the movement of irregular migrants, including asylum seekers.
- Some strategies, such as immigration detention, pose a serious threat to health and mental health. Others, such as the use of visa restrictions or other pre-emptive interception measures, have a potentially large impact on migrants' health and welfare by forcing people to remain in settings where they face the chance of persecution.
- Interception can also promote humanitarian outcomes. Refugee camps, for example, address immediate protection, safety, and service needs of forcibly displaced persons, but they have limits as long-term solutions.
- Migration interception practices are a major global determinant of health and mental health. Health professionals must remain engaged in discussions about migration and humanitarian protection to ensure a broader consideration of the health impact of these practices.

**This is one article in a six-part** ***PLoS Medicine*** **series on Migration & Health.**

## Global Immigration Policy Environment

Determining who may or may not enter and remain within a set of territorial boundaries is a defining feature of the modern nation state. In the current era of globalization, the sheer volume of international travel—for business, tourism, employment, and education, as well as permanent resettlement—places formidable pressure on states attempting to manage population flows. Unlike trade, monetary, or health sectors, where formal multilateral frameworks are regulated by governing structures such as the World Trade Organization (WTO), International Monetary Fund (IMF), or the World Health Organization (WHO), no internationally agreed policies exist in relation to global immigration. Hence, immigration policy responses are determined by the state and reflect the perceived impact of immigration on multiple intersecting factors, such as economic and political opportunities, social cohesiveness, national identity, and foreign policy interests.

The exception to this principle is that the state and its nominated agents have an international duty under the United Nations (UN) Refugee Convention (see http://www.unhcr.org/) and the Convention Against Torture to ensure that no one who seeks protection is forcibly returned or *refouled* to a country in which they may face persecution or torture [1],[2]. Other challenges to state control emerge from growth in irregular migration; that is, the travel of persons to or within a state's territory without authorization. Irregular migrants variously comprise asylum seekers, those looking for employment or family reunion, and victims of human trafficking [3]. The confluence of low-skilled economic migration and forced migration due to humanitarian crises, often from the same regions, results in immigration policy being a highly sensitive policy area for many states.

Health is rarely considered as a driver of immigration policies, but the health impacts of strategies to manage immigration are becoming well documented [4]–[6]. State interception policies designed to halt and limit the flow of irregular migrants in particular raise significant health concerns. It is important for health policy-makers and public health professionals to be aware of the policy pressures leading to the implementation of such measures and, where possible, to find ways to ameliorate their impact or advocate for alternative approaches.

## Strategies to Manage Migration

The targeted application of entry visa restrictions represents the primary strategy applied by states to meet immigration policy targets and to limit access to their territory [7]. Visa restrictions are routinely applied to nationals from countries seen to be at high risk of generating irregular migration [8]. Substantial disparities in freedom of travel result; for example, nationals from Afghanistan, Iraq, Iran, Somalia, and Sudan, all major refugee-producing settings, are amongst the most visa-restricted globally [9]. In high-income settings, visa restrictions are often coupled with other visa enforcement measures, including the imposition of heavy fines on carriers transporting passengers without valid documents or the dispatch of immigration officials to ports of departure to undertake document inspection. Some strategies directly involve health services, for example, through the use of medical screening to exclude migrants and refugees with communicable diseases such as hepatitis B, tuberculosis, and HIV, or to prevent those with existing illnesses from migrating, ostensibly in an effort to reduce the potential burden on health services within the host country [10],[11].

Some possibilities for realizing health gains in circumstances where interception strategies are unlikely to change may be present [12]. For example, the sheer number of health checks undertaken annually has enabled participating organizations to use screening data to support public health surveillance programs in source countries and to facilitate access to treatment [10],[13]. Nevertheless, collectively these approaches extend migration control beyond the immediate borders of the state. Many states also apply safe third country provisions, in which asylum seekers are refused admission to a state's territory or asylum procedures if they have passed through a territory where it is deemed that they could have sought protection. The United Nations High Commission for Refugees (UNHCR) has raised concern about such provisions, especially if the asylum seeker is not given the opportunity to address the presumption of safety in the transit country [14].

Numerous global health risks arise from the widespread use of such measures. Most direct is the demonstrably increased risk to the physical safety of vulnerable populations prevented from fleeing situations of persecution, violence, and dislocation. Adverse mental health outcomes are well documented with a clear link between the level of political violence in a country, exposure to human rights violations, and the prevalence of posttraumatic stress disorder and depressive disorder [15]. These pre-emptive interception measures also have the outcome of creating an incentive for asylum seekers and other irregular migrants to bypass immigration controls, often involving the use of human smuggling networks [8]. It is estimated that as many as 4,000 people lose their lives annually from drowning, suffocation, heat exposure, or other adverse events from attempts to enter Europe, Australia, and North America clandestinely [7],[16]. Many states now routinely intercept vessels suspected of carrying asylum seekers, and while there is some evidence that this form of interception may disrupt irregular migration networks [7], increasing reliance on interdiction will likely generate more hazardous smuggling networks and push asylum seekers on to other states [8],[17].

From a policy perspective, the implementation of stringent visa restrictions and other pre-emptive interception measures does little to address the population pressures leading to the growth in irregular migration [17]. Whilst the greatest source of refugees in recent decades have been amongst African states, the resulting displacement has primarily been to surrounding African nations with humanitarian relief efforts contained within those settings [14]. This can be contrasted with other areas of conflict, where displaced populations are more likely to seek protection through permanent resettlement under the provisions of the UN Refugee Convention.

Over the previous two decades, the majority of permanent refugee resettlement places have resulted from asylum seekers reaching the borders of recipient countries, in large part via irregular migration, and applying for protection [14]. An alternative protection model involves the use of planned resettlement directly from conflict-affected regions. During 2009, extraterritorial assessment and processing undertaken by UNHCR or other agencies resulted in the resettlement of 112,400 refugees or “persons of humanitarian concern,” primarily to the United States, Canada, and Australia, countries that all have annual off-shore humanitarian programs. Commitment to planned refugee programs has been an area of neglect, especially in the European Union (EU), where there is no substantive tradition of off-shore humanitarian resettlement. Recent developments within the EU to harmonize and unify immigration and asylum procedures between member states may provide a framework for advocating for the development of such a program [18],[19]. It is important to note, however, that threats to health and well-being can also arise from extraterritorial processing in situations where it is not possible to meet the demand for resettlement places. Refugees waiting for resettlement face a range of health risks associated with the difficult and possibly insecure nature of their living environments [20] (see Humanitarian Interception section below). This situation reached a crisis during the late 1980s with the pending resettlement of Southeast Asian refugees. The breakdown in a global commitment to resettlement led countries in the region to transition makeshift refugee encampments to secure detention facilities as part of their own border protection measures. Since this time there has been a growth and commitment to the use of immigration detention as a key interception strategy, a development that raises significant health and human rights issues.

An important recent example of effective multilateral burden-sharing occurred in response to the Kosovo crisis and the international humanitarian intervention following Serbian military aggression. At the height of this crisis in 1999, some 800,000 Kosovar Albanians fled or were expelled from Kosovo to the surrounding states of Albania, the former Yugoslav Republic of Macedonia and Montenegro. Under the Humanitarian Evacuation Programme (HEP), 92,000 Kosovar Albanian refugees were transferred under temporary protection provisions to 29 host countries [21]. This effectively averted a humanitarian disaster in the region and allowed for a multi-sectoral response to the humanitarian needs of refugees, including the provision of comprehensive health care. The temporary nature of the resettlement made the model attractive to recipient countries. In most cases, Kosovar Albanians wanted to return home once safe to do so, with many also committed to building a future independent state. The expectation of return can be difficult, especially for those with traumatic stress reactions or other health problems. In general, the use of temporary protection appears to be a viable solution only when there is a clear commitment by recipient countries to return evacuees once safety has been fully restored to the source country. The use of temporary protection provisions for populations who remain at risk of persecution, as occurred with refugees arriving through irregular migration channels in Australia [22], appears to be a highly counterproductive approach that leads to adverse mental health outcomes that persist until permanent protection is granted [23],[24].

## Interception and Confinement

Detention of irregular migrants—including asylum seekers—now comprises a routine component of the migration control mechanisms of multiple countries in the developed and developing world [25] (see Box 1). Numerous breaches of human rights have occurred in immigration detention settings across multiple jurisdictions [26],[27]. By its very nature, the practice of immigration detention represents a threat to key human rights principles: the right to seek asylum; the right to be free from arbitrary arrest, detention, or exile; freedom of movement; and the right to be free from torture or cruel, inhumane, or degrading treatment or punishment [28]. There are often no special provisions to support vulnerable populations such as torture and trauma survivors. Women and children are often detained in the same settings as single men, exacerbating situations of vulnerability. The Convention on the Rights of the Child (http://www.unicef.org/crc/) explicitly states that detention must be a measure of last resort and for the shortest appropriate period of time, yet children are routinely subject to various forms of detention [29].

**Box 1. Immigration Detention Practices**

Detention may be implemented at different stages of the migration cycle. In the United Kingdom, for instance, detention tends to be applied as part of removal procedures for asylum seekers whose claims have not been upheld. Most commonly, however, immigration detention occurs at the point of arrival or interception. In situations of mandatory detention, such as that applied in Australia since 1992 and increasingly in other states, detention is not predicated on a merits-based assessment (such as the likelihood of absconding or suspected criminal intent) but follows automatically from the mode of arrival. Detainees are generally denied the right to appeal to an independent judicial body or tribunal to challenge the fact of their detention. Where it is not possible to effect repatriation, detention can occur for long periods, with some detainees being held in the Australian detention system for up to 7 years. The settings of detention vary from state to state. In general, special purpose detention facilities are used, although in United States, the majority of persons subject to immigration detention are held in state prisons, often with no practical separation from the convicted population [56]. There is a strong international trend for detention facilities to be located in remote or non-urban areas, thereby limiting access to representation and services. There is also an increasing trend for high-income countries to provide funding for extraterritorial detention, with a number of European states providing financial support for a string of detention centres across the North African and Sahel states [67]. Similarly, the Australian government has provided funds to support the construction and operation of a series of detention facilities throughout Indonesia, Malaysia, and other countries within the Asia-Pacific Region.

From the outset, health and other aid workers expressed significant concern about the impact of detention on asylum seekers in Southeast Asia [30],[31], the United Kingdom [32], Australia [33],[34], and the United States [35],[36]. Mental health research indicates that rates of mental disorder amongst populations held in detention are substantially higher than compatriots held in community settings [22],[37]–[40]. Children in particular show evidence of severe mental health impairment [41]–[44]. Rates of suicide and self harm have been documented as being many times higher than in community settings and at a level comparable to or higher than that amongst prison populations [45],[46]. Prospective research documents a pattern of deteriorating mental health as length of time in detention increases [42],[47]–[49]. Studies surveying refugees released from detention suggest that the practice may result in prolonged mental health impairment [37],[50],[51].

Collectively, the evidence from all sources [52],[53] suggests that immigration detention represents a major threat to the mental well-being of displaced populations in the short and long term. The concerns raised by health professionals have gone largely unheeded by governments, and the practice of mandatory detention has continued to expand globally. There has been an evident attempt by many states to increase the availability of health and mental services within detention [25],[54]. Despite this, general health outcomes within detention are poor, particularly in regard to the management of chronic conditions [27],[55],[56]. Immigration detention provides a clear example of the intrinsic link between the denial of basic human rights and deterioration in health, and mental health in particular [26]. Confinement, isolation, lack of freedom, perceptions of being arbitrarily punished, uncertainty about the future, and fear of being returned to situations of danger all converge to create a pattern of deteriorating mental health [49],[50] that does not appear to be evident in community-based alternatives [22],[37],[57]. The mental health cost to clinicians working within detention facilities has also been substantial, with documentation of high levels of secondary traumatisation [54],[58],[59]. This situation has led some humanitarian organizations to remove their services from detention settings [60], and a number of professional bodies [27] have made cautionary statements to their members. Health providers in such settings must be aware of the complex dual loyalty conflicts that can arise [58],[59]. At the very minimum, health workers and policy-makers need to ensure that health services within detention maintain an actual (in terms of organizational structure) and an effective (in terms of clinical decision making) autonomy from detention operating authorities. It is imperative that the health community continue to raise awareness about the harmful nature of this form of interception and to advocate for a return to less restrictive alternatives such as the use of open residential facilities or other forms of community integration during refugee processing [54],[61].

## Humanitarian Interception

Low- and middle-income countries continue to absorb the vast majority of forced migrants, hosting an estimated 8.3 million refugees, or 80% of the global refugee population, during 2009 [62]. Populations displaced to surrounding countries or held in situations of transit place a heavy burden on developing nations. Refugee camps provide a form of interception response that aims to address the immediate protection, safety, and service needs of forcibly displaced populations. Much has been documented concerning the health consequences of camps as a model of interception, emphasizing the associated benefits and dangers for protection and service delivery [20],[63],[64]. Most of the health risks relate to the consequences of confining populations in impoverished settings, especially when compounded by poor sanitation, hygiene, shelter, water quality, nutrition, and security. The leading causes of mortality in such camps are related to malnutrition and communicable disease [65]. During acute emergencies, up to 40% of deaths may be attributed to diarrhoeal diseases, most prominently due to the cholera and *Shigella* pathogens [64], with measles also contributing to high child mortality [20]. Malaria, acute respiratory infection, tuberculosis, and meningococcal meningitis may all pose serious threats, depending on the area of displacement [20],[66]. Many of these significant public health issues also pose serious problems for the host population. Careful selection of sites and consultation with local service providers may enhance the scope for improving food security and health programs for both refugees and the surrounding, often also under-served, population [20].

Nevertheless, there has been substantial debate about whether refugee camps remain the preferable response in managing forced displacement and other complex emergencies. Some commentators such as Harrell-Bond [63], a leading researcher on forced migration, argue that camps are of little benefit to the affected communities or the host population, but exist primarily to make it easier for service providers. In particular, the refugee camp environment may undermine key aspects of family life by failing to provide opportunities for gainful activity or employment and by fostering dependency on relief aid. Humanitarian operations within camps are also likely to bypass local institutions by importing external expertise or, at times, may contribute to the weakening of host country institutions by attracting local staff to international agency positions with higher salaries. This has led to the establishment of refugee camps themselves being questioned, especially in situations where camps have become semi-permanent. UNHCR defines such protracted refugee situations as ones in which 25,000 or more refugees have been in exile for 5 years or longer [62]. Based on this definition, it is estimated that there were 5.5 million refugees in protracted situations in late 2009 [62]. Hence, while refugee camps may remain an important tool of humanitarian interception during the acute phases of complex emergencies, they also pose health risks in the medium to long term. This appears to be reflected in higher rates of depression in these settings compared to populations that have been resettled to third countries [15]. As with immigration detention, a rights-based approach to health can assist in identifying contextual restrictions and abuses that pose significant risk to health and well-being in these settings [26].

## Concluding Comments

What appears to be consistently missing from interception policy responses is an adequate recognition of the health impacts of different forms of interception. There is clear evidence now that some strategies, such as the use of immigration detention for processing asylum seekers, represent a serious threat to health and mental health in particular. Other practices, such as pre-emptive interception, have a hidden but possibly large global impact on health and welfare by forcing people to remain in settings where they face the real chance of persecution. It is vital, therefore, that health professionals remain engaged in discussions about migration, and humanitarian protection in particular, to ensure a broader consideration of the health impact of interception and to advocate for a global response that promotes health.
